# Covid-19 testing in Primary Health Care: professionals’ perceptions

**DOI:** 10.11606/s1518-8787.2026060006430

**Published:** 2026-05-01

**Authors:** Denise Nogueira Cruz, Sandra Garrido de Barros, Ana Clara de Rebouças Carvalho, Thais Regis Aranha Rossi, Laio Magno, Inês Dourado

**Affiliations:** I Universidade Federal da Bahia. Faculdade de Odontologia. Departamento de Odontologia Social e Pediátrica. Salvador, BA, Brasil; II Universidade Federal da Bahia. Instituto de Saúde Coletiva. Salvador, BA, Brasil; III Universidade do Estado da Bahia. Departamento de Ciências da Vida. Salvador, BA, Brasil; IV Fundação Oswaldo Cruz. Instituto Gonçalo Moniz. Salvador, BA, Brasil

**Keywords:** COVID-19, Primary Health Care, Health Services, Testing

## Abstract

**OBJECTIVE:**

To analyze the perceptions of Primary Health Care (PHC) professionals regarding Covid-19 testing in basic health units (BHUs).

**METHODS:**

This qualitative study, part of a formative research project, involved 22 semi-structured interviews and 4 focus groups with professionals working in PHC in a Brazilian capital city. Among other criteria, the professional most directly involved in pandemic response measures in each BHU was selected. Content analysis was performed using a deductive analytical approach, in which the findings were organized into categories defined based on the components of the health system (population; infrastructure; organization of services; service delivery or care model; and management).

**RESULTS:**

Reports commonly referred, in the population component, to the challenges arising from the limitations of PHC coverage; in the infrastructure component, the insufficiency of human resources and physical infrastructure was highlighted, which in turn required changes in the organization and provision of services, with repercussions on the care model. The contributions also revealed that at some points during the pandemic there was a disruption of the work process, especially in family health teams. In the management component, weaknesses in communication flows between the health secretariat and the BHU were identified, although this was less frequently mentioned.

**CONCLUSION:**

The perception that expanding and decentralizing testing in BHUs was necessary was confirmed. The findings point to the importance of coordinating the pandemic response and effectively decentralizing the actions adopted, of preparedness plans and strengthening PHC and its professionals, as well as investments in infrastructure and team training, and advances in actions aimed at occupational health. The pandemic revealed the importance of advancing the health economic-industrial complex and, fundamentally, strengthening and defending the Brazilian Unified Health System.

## INTRODUCTION

The Covid-19 pandemic had repercussions in different areas of human life and required changes in the organization and provision of health services on a global scale. The initial absence of vaccines and other pharmacological measures implied the adoption of containment and mitigation actions to be complemented by testing and screening strategies^1^. However, testing and diagnosis, in the early stages of the pandemic, represented a challenge due to difficulties that included aspects related to the biological material to be used, the methodology to be employed, the accuracy of the tests, and the availability of supplies and resources given the global demand^2^. Thus, the development and availability of rapid antigen tests constituted a strategy to expand access to testing and combat the pandemic by making it possible to diagnose active cases and prevent the spread of the virus^3^.

In the initial context of the pandemic, the Brazilian federal government prioritized testing in hospitals and health facilities for symptomatic individuals^4,5^, and the absence of a coordinated and comprehensive national strategy for testing and tracing accentuated the challenges of supplying diagnostic inputs and containing the spread of the virus^4,6^. On the other hand, the articulation of local governments and other social actors around initiatives to increase testing capacity and national production of components stands out, such as the improvement of PCR tests; the development of technologies; and the implementation of regulatory measures to accelerate new tests^4^.

In Brazil, the existence of a public and universal health system, the Unified Health System (SUS), was fundamental in facing the pandemic^7^ — which required the (re)structuring of specialized care, such as the expansion of hospital beds, but also of Primary Health Care (PHC). However, as in other countries, there was a low prioritization of PHC^5^, which, despite having played a relevant role^8^, may have had its potential limited, given its capillarity throughout Brazil. The broad coverage of PHC would likely enable the strengthening of surveillance measures in the response to Covid-19, based on a community and territorial approach, and would allow for the enhanced performance of community health workers (CHWs), especially in situations with limited diagnostic and testing capacity^5^.

The fragility in the coordination between health surveillance and PHC in the Brazilian response, and the low levels of testing during the initial period of the pandemic, hindered monitoring of the epidemiological situation^7^. Regarding health surveillance actions, an investigation in large municipalities in Northeast Brazil found incipient actions^9^, while in a study with basic health units (BHU) across the country, the worst results, in all five Brazilian regions, were related to the collection of diagnostic tests^8^.

On the other hand, there are strategies with positive outcomes, such as the initiative of municipal managers and PHC managers to establish regional coordination and partnerships with universities, which enabled increased testing^10^.

During the pandemic, the practices of PHC professionals were modified, and the literature has studies on these professionals’ perceptions of what are understood as crisis situations and how to manage them^11^, on the role of PHC in confronting the pandemic^12^, and regarding the Covid-19 vaccination campaign^13^. However, to our knowledge, the perception of professionals regarding Covid-19 testing has not been previously explored. The end of the pandemic state of emergency was declared in May 2023^14^, and the accumulated evidence calls for investigations that contribute to evaluating responses in the management of future health crises.

Thus, the objective of this study was to analyze the perceptions of professionals working in PHC regarding Covid-19 testing in BHUs of a Brazilian capital city.

## METHODS

This was a qualitative study using data from the formative research (FR) of the project "Expansion of testing, quarantine, e-health and telemonitoring strategies to contribute to the fight against the Covid-19 pandemic in Brazil — TQT Covid-19", developed in two Brazilian municipalities. FR sought to understand the work process in basic health units (BHU) — with and without Family Health Strategy (FHS) as well as the challenges related to conducting Covid-19 testing in these units, to identify more appropriate strategies for their implementation and expansion. Although conducted in both municipalities, this study focused on the data from FR of one of the municipalities.

Twenty-two semi-structured interviews and four focus groups (FGs) were conducted with 23 professionals from different categories, working in one of the 22 BHU with and without FHS involved in the study located in a populous territorial region with high social vulnerability in a Brazilian capital city. Considering the participation of two professionals in both strategies (FG and interview), the study involved 43 professionals.

The data were collected between December 2021 and March 2022, noting that during this period, testing was not performed in all BHUs. The interviews were conducted in person and individually with one professional from each selected BHU, based on: the indication of the BHU management (convenience); availability; direct involvement in actions to combat the pandemic (intentionality); and at least one year of experience in the position held (eligibility). The FGs were composed of professionals from some BHUs that performed Covid-19 tests and others that did not, and by the respective BHU managers. One FG was formed only by CHWs from different BHUs. The interviews and FGs were conducted by researchers who were part of the FR team — and in the FGs, specifically, more than one researcher was present to ensure moderation and support in conducting the study.

The interview script addressed issues related to the Covid-19 pandemic: containment measures and the role of PHC; the organization of testing, vaccination, and case surveillance actions in BHUs; knowledge about Covid-19 tests; social protection measures; and sources of information/news about the pandemic. The FG script had Covid-19 testing as its central theme, including questions about procedures, criteria, and difficulties in performing tests in BHU; training and knowledge of professionals, seeking, through group interaction and different points of view, to understand perceptions and practices surrounding testing.

The interviews (ranging from 29 to 104 minutes) and the FGs comprised respectively 23 hours and 19 minutes and 5 hours and 47 minutes of recordings that were transcribed in full, reviewed, and coded. After transcription, content analysis was performed, including the stages of pre-analysis, material exploration, and treatment, inference, and interpretation of the results^15^. The analysis aimed to understand perceptions about testing in BHUs, articulating them with the components of a health service system or network^16^.

The study sought to understand the implementation of testing through professional accounts; through "concrete stories"^17^, aware of the fact that they bring lived experience as subjective experience, given their different trajectories and relationships with the event^18^.

The analysis was conducted according to categories on the components of a health system adopted by Souza and Bahia^16^, with five of the components being applied (population; infrastructure; organization of services; provision of services or care model; and management), as shown in Chart 1. On the basis of the constituent elements of the health system components, the following analytical categories were established: population under care/enrollment; human resources/workers; inputs; physical structure/BHU; knowledge/skills about testing; assistance, surveillance and continuity actions of PHC programs; logic/model of organization of actions in BHUs; standardization/regulation of practices; and elements of macro- and micro-management.

**Chart 1:** Summary of the characterization of the components of a health care system or network adopted by Souza and Bahia^16^ and used for data analysis.

HEALTH SYSTEM OR HEALTH SERVICES NETWORK	COMPONENTS
1) Population	Populations that are under the health responsibility of the health care networks; of the health teams
2) Infrastructure	It includes various types of resources that can be classified into: Health workers; Health facilities/physical infrastructure of the health network; Medicines, equipment, and other supplies; Knowledge (of the health status of people, of technologies for intervening in health and disease, and of the functioning of services).
3) Organization of the health care services	How human and technological resources are organized to carry out health actions. This includes, within the scope of the Brazilian Unified Health System (SUS): Health assistance; Health surveillance; Special policies and programs; Policy for humanizing health care.
4) Provision of services or health care delivery model	It includes actions for health promotion; prevention, diagnosis and treatment of diseases and health problems; and health rehabilitation. They can be developed on the basis of different logics or rationalities, configuring different care models: Hegemonic model (privatized medical-assistance model); Public health model; Alternative models (e.g., health surveillance, health promotion and healthy cities, a model in defense of life, and programmatic health actions, among others).
5) Management	Management is articulated with the concepts of governance ("the act of governing well") and regulation (laws, rules, norms, and instructions). Management can be categorized according to: Levels of management: macro-, meso-, and micro-management; Dimensions: political, technical, and administrative (at each level)).

Note: the authors also adopt a sixth component, funding (not applicable in this study).

Source: Souza and Bahia – summary of the text presented on pages 53–60^16^.

The project was approved by the Research Ethics Committee under No. 53844121.4.1001.5030 and by the Research Ethics Committee of the World Health Organization (CERC.0128A and CERC.0128B), and the professionals agreed to participate by signing an informed consent form.

## RESULTS and DISCUSSION

The study involved 43 professionals, most of whom were female, self-identified as Black or mixed-race, nurses, and between 30 and 45 years old.

### Population

According to Souza and Bahia^16^, the population is the main component of a health system and should be territorially defined so that health networks and teams (especially those of the FHS) respond to health needs and assume health responsibility for that population.

The results obtained revealed that the participants perceived how important population assignment and territory definition are in the organization of services in PHC.

"[…] the demand is very high, and not all units are performing the tests. […] *all units should be performing the tests. Even considering the ease of access to the unit* […] *the patient often walks to the unit*. They don't come by public transport; they don't have the money to pay for a bus ticket. *So, if it's close to home, it's easier.* Many people don't get tested because they can't get there. […] So, many people go through the illness without even being seen, without even receiving assistance" (Interview-BHU19).

For health care professionals in teams with and without FHS program, conducting tests in all BHUs would improve service delivery and reduce the barriers faced by the population.

"[imagine] I suspect I have Covid, I'm isolated at home, *but there's a health center here in my neighborhood nearby. I'll go there, get tested, and go back home*. But no… There's only that one health center far away that I have to take transportation to […] and that's already contaminating other people, right? […] There should be more testing units. Every neighborhood should have a testing site to make it easier for people and so they don't have to travel far." (Interview-BHU20)

PHC is preferably the first point of contact for individuals with the health system, acting as the communication center of a care network and organizing the care provided^19^. Integrated health systems^16^ that strengthen PHC with the definition of an assigned population are more effective^19^. The organization of PHC with the implementation of adequate and sufficient BHU, offering services that meet the needs of this population and that are geographically closer, can reduce financial, geographical, and organizational barriers^20^, such as those perceived by the interviewees.

### Infrastructure

It is the set of resources necessary for the provision of services, including: health care workers (considering quantity, skills, distribution, training, and working conditions); health network facilities/physical infrastructure; medications/equipment/supplies; and knowledge (of the population's health status, technologies, and the functioning of services)^16^.

In the domain of the "infrastructure" component, the reports indicated that ensuring testing in BHU faced challenges related to the four types of resources (workers, supplies, physical structure, and knowledge). The findings and analysis on infrastructure (for each type of resource) are presented below, and excerpts from the statements are consolidated in Table 2 at the end of this component.

The insufficiency of workers, perceived both in the BHU that performed testing for Covid-19 and in those that did not, was associated with the challenges of incorporating testing and guaranteeing other actions inherent to PHC, as revealed in Statement 1 (S1) in Chart 2. The challenges, in addition to the quantity of human resources, also included, for various reasons, the absence of certain professionals (S2, S3 — Chart 2).

**Chart 2:** Excerpts from individual and focus group interviews related to the categories established for the infrastructure component.

Resource – Workers	Statement 1 (S1)	"[…]The issue of having testing here is a *personal matter; we don't have the human resources for it*. […] If we add another thing, we might not be able to handle it. […] If we had testing, we wouldn't have the vaccine. […] We need to consider the professional who performs the tests, their training, who can do it, the service hours, whether it would need to be every day. We would have to develop a whole strategy." (Interview-BHU5).
	Statement 2 (S2)	"[…] One of the major difficulties we faced when we started testing here was precisely the *lack of a doctor* to prescribe and refer patients." (Interview-BHU18).
	Statement 3 (S3)	"[…] this past week […] the unit had to close and testing was suspended. *This was due to staffing issues*. The team […] *tested positive.* […] there were no conditions to replace the nurses, no way to replace the cleaning staff. […] testing is suspended until further notice" (Participant5-FG02).
Resource – Supplies	Statement 4 (S4)	"[…] *I think that if more tests were available, we could meet a greater demand* […] because if we don't receive them, then we end up really limiting […]. *Many people even have the symptoms, but they don't go to the health units to get tested because of the difficulty in obtaining a test*, because sometimes the unit only performs a certain quantity, and so on, and then the person doesn't seek the service and just thinks: ‘I'll treat myself at home’" (Participant4-FG04).
	Statement 5 (S5)	"It's distressing! You see a line of 30, 40 people and only being able to do 12 tests is distressing!" (Participant1-FG01).
	Statement 6 (S6)	"For people to have access, what did they [referring to the municipality] implement […]? They removed the testing […] from inside the health units, right? To put it in these mobile locations, right? […] *It's not in a health unit, it's on the street, right? A mobile unit goes there*… It arrives, stops, and performs the testing." (Interview -BHU20).
	Statement 7 (S7)	"Expand or make available in almost all locations, like the rapid testing for sexually transmitted diseases. *So, it could be available in all health centers, right? Covid testing.* And, during the peak of the pandemic, *disseminate it to supermarkets and shopping malls*." (Interview -BHU12).
Resource – BHU infrastructure	Statement 8 (S8)	"Here, we didn't want to treat symptomatic patients *because of this. Because you're mixing two groups of people*. You're mixing healthy people with sick people. […] a person who comes here healthy to get vaccinated ends up crossing paths with someone who already has symptoms and runs the risk of leaving here sick. *We don't have the physical infrastructure for that. If it were a larger unit, with two doors, one entrance and one exit*…" (Interview-BHU05).
	Statement 9 (S9)	"[…] *the right thing to do wasn't to have it inside a basic health unit.* […] how are you going to put a symptomatic patient in a unit where there's a pharmacy, where there's vaccination, where there are […] all kinds of services […]. *How are you going to get in line at the pharmacy and explain to people* […] *that that corridor is a risk zone, that it's for Covid,* you understand?" (Interview-BHU06).
Resource – Knowledge	Statement 10 (S10)	"[…] as far as I know, *none of us were trained* for either of the two tests [PCR and rapid test] […]. When it started, […] none of us were the ones doing it" (Interview-BHU01).
	Statement 11 (S11)	"[…] we learned through trial and error […]. We didn't receive any training […]. In my unit, we would sit down and talk, right? […] but, *personally, I didn't go through any training*" (Participant3-FG03).
	Statement 12 (S12)	Participant 2: "I was [trained] by her […]. *She was my teacher*." Participant 5: "Not me, *the colleague who learned it taught me*." Participant 4: "*Only a few technicians*, and we have a woman who works in the laboratory. Only they received the training." (Participants 2, 4, 5-FG01)).

BHU: basic health unit; FG: focal group.

The limitation of supplies restricted the performance of tests and was also perceived as a factor resulting from the population's lack of demand for the service and generating anxiety among the interviewees (S4, S5 — Chart 2).

It is also important to observe in S4 the contrast with the insufficiency of workers. Another perception of the testing experience in BHU emerges, with the availability of tests prevailing as a challenge.

The anxiety expressed in S5 reflects the suffering of the professional resulting from a demand for tests greater than the availability in the BHU. The pandemic, for various reasons, triggered mental illness in health professionals in PHC^21^. These findings should prompt the development of robust programs aimed at worker health and not only in this municipality.

A recurring perception was the need to decentralize testing to all BHUs and other social spaces (S6, S7 — Chart 2); a measure that would expand the population's access to diagnosis, but which had a considerable limitation in the insufficiency of supplies.

The scarcity of Covid-19 tests on the international market in the early stages of the pandemic resulted in the prioritization of testing and the implementation of strategies such as "testing centers" and the drive-through system — these also incorporated in South Korea^22^. This country, like China, was successful in its response to the pandemic; both relied on non-pharmaceutical measures and invested in expanding testing^22,23^.

The limitations of the physical infrastructure of the BHU were described by many interviewees as obstacles in guaranteeing the simultaneous provision of tests and vaccines for Covid-19 (S8, S9 — Chart 2). The size of the BHU, internal circulation space, sizing of user entry and exit flows, and the positioning of consulting rooms, sectors, and the pharmacy are some of the difficulties pointed out that require attention from managers in the planning and construction of facilities, anticipating future health crises.

Considerations about the infrastructure of the BHU and the importance of their location being close to their respective assigned populations raise reflections on the challenges related to the geographical territory, especially in densely populated areas of large urban centers. Guaranteeing area for the installation of territorially and structurally adequate BHUs becomes a challenge, in addition to human and financial resources, which must be addressed in different forums — such as those discussing municipal development plans.

Regarding knowledge, another type of resource, the reports on professional training for conducting tests in BHU frequently mentioned the processes of ad-hoc training or even the lack of any training at all (S10, S11 — Chart 2), as well as the strategy of replicating instructions by a trained colleague (S12 — Chart 2). However, most felt prepared to perform the testing.

It should be noted that the municipality provided in-service training for rapid antigen testing through professionals from the municipal central laboratory, an aspect indicated in F12. The statements, however, did not mention the use of digital technologies or training strategies specifically related to testing — a fact that is noteworthy, given the expansion, since the pandemic, of the use of new digital technologies with applications, for example, in health surveillance^24^.

### Organization of Health Services

This component defines how health care assistance, health surveillance, special policies and programs, and the humanization policy are organized, based on available human and technological resources^16^.

It is important to note that the limitations involving the components of the health system presented so far, especially the infrastructure of the BHU, generated perceptions regarding the dimensions of service organization and also the provision/model of care (as observed in the reports below).

The challenges of PHC coverage, the infrastructure of the BHU, and the insufficiency of tests/supplies negatively impacted health care actions for the population and the continuity of previously implemented programs and were also perceived as obstacles to articulation with health surveillance actions.

"[…] *We don't have good primary care coverage, so logically we won't have good testing coverage […] in my opinion, testing isn't just for patients who have symptoms.* We test symptomatic patients, but those who are asymptomatic are still spreading it to everyone" (Participant1-FG4).

"[…] we concentrate too much […] and we don't distribute the work effectively. *This generates confusion for those who have a work process in place* […] the idea is that the Covid-19 actions, over time, should become part of the teams’ work process. So, when you concentrate the work, the team, in a way, slows down its work process, and this penalizes the other tasks that the team was already working on. *So, if you distribute the work, you can reduce this overload, and the teams can continue with their other work*. *Because in the unit that does testing, then for this, for that* […] you penalize that unit that is providing the service even more" (Participant 2-FG4).

"*I have to have the test done […]. Without the test, I don't have a clear picture […] and what gives me the basis to intervene is epidemiological investigation.* If I don't do epidemiological investigation, I'm just relying on my own assumptions. And, based on my assumptions, I'll only address isolated outbreaks and won't put out the entire fire*"* (Interview-BHU21)).

### Care Model

It refers to the "combinations of material and immaterial technologies used in interventions on health problems and needs"^16^. It comprises hegemonic and alternative models^25^.

It should be noted that the understanding here is that the guiding logic of the practices of PHC teams, especially the FHS, should be towards "alternative care models^25^". As a proposal for redefining practices and reorganizing services, the care model Health Surveillance advocates the articulation of actions to control determinants, risks, and damage to health, with an individual and collective approach, and action in specific territories, to address problems and improve the living conditions of the population^25,26^. This model overcomes the dichotomy between epidemiological and health surveillance ("collective practices") and outpatient and hospital care (individual practices), integrating them and also enabling the articulation of spontaneous demand with the supply organized by population groups in the conception of programmatic health actions^26,27^.

In the course of this discussion, it stands out that the professionals’ accounts revealed dilemmas and tensions regarding the perception of: the decharacterization of practices during the pandemic, especially in the initial phase and during periods of recrudescence of cases; the discontinuity in the monitoring of certain programs; and the absence of a "programmatic" agenda.

"During this most intense period of caring for patients with respiratory symptoms, we were practically *closed to internal program demands* […]. So the unit's space was focused on this demand […] which was caring for patients with respiratory symptoms" (Interview-BHU14).

"[…] in fact, I only saw Covid! After a while, I started seeing chronic diseases […] *chronic diseases that had lost follow-up* because the outpatient clinic was suspended […]. So, chronic diseases that were under control, that were compensated, were becoming decompensated in an overcrowded system" (Interview-BHU17).

"*Elective procedures were practically suspended*… We continued treating tuberculosis […]. If it was a hypertensive or diabetic patient with decompensation, everyone could be seen […]. During the waves, in all the ups and downs of the waves, we lived like that. Whenever we saw that the number of symptomatic patients decreased, then *we would reduce the shifts of the team exclusively for symptomatic patients and open the schedule*. So we lived like that, we still live like that, right? […] we live observing the wave […]. *So, whenever it increases, we get tense: ‘We'll have to change […]’. We live in tension all the time*" (Interview-BHU03).

The pandemic rapidly affected health systems, impacting the work processes of primary health care teams, as the findings above indicate. The impacts on PHC were observed in the different phases of the pandemic^28^, such as the initial suspension of essential and routine activities and, subsequently, the resumption of some practices combined with the incorporation of new actions — for example, testing — leading to a disruption of the PHC work process^28^.

### Management

This component refers to how, at different levels (macro-, meso-, and micro-management), actions and formulations are developed in the political, technical, and administrative dimensions of health systems^16^. Management is articulated with the concepts of governance and regulation.^16^

The management component was occasionally mentioned, but aspects related to regulation, specifically the definition of norms and instructions or even the fragility of these mechanisms, were noted (S13 — Chart 3).

**Chart 3:** Excerpts from individual and focus group interviews related to the categories established for the management component.

Management – standards/regulation	Statement 13 (S13)	"[…] if there is a pandemic, we know that we will have to provide care, *that this protocol should be in place from the moment patients arrive*. We only received the guidelines three months later. ‘Oh, it caught everyone by surprise.’ I think that, in healthcare, certain things shouldn't be a surprise. […] what I wanted, from the entrance to here: the responsibilities of each person. […] we were the ones who came up with everything! *Three or four months after it arrived to us*." (Participant1-FG01).
Micro-management	Statement 14 (S14)	"[…] total chaos […]. That's what I was saying: *they crossed the street, and the unit is completely different*" (Interview-BHU03).
	Statement 15 (S15)	"These are different types of services […]. It ended up looking like this: *that we implemented a war strategy* […] and we experienced something we had never experienced in our lives; nobody had ever experienced a pandemic. So, *it would be very strange if the colleagues at one health center acted the same way as those at another health center*" (Participant 6-FG04).
	Statement 16 (S16)	R: […] each unit has its own dynamics. But, *at a minimum, this needed to have a standard, right?* It doesn't. P: […] I remember the meeting […] they said that one unit did 40 tests, another did 50, another did 10. Who determines that? R: Nobody. It's the unit. The unit, in dialogue with the [refers to regional management], determines it […]. *It has no relation to demand whatsoever*. The municipality leaves it up to the teams. […] here we had several tensions" (Interview-BHU03).
Macro-management	Statement 17 (S17)	"[…] what makes things difficult in the municipality […] is not only access to testing but also access to primary care and health care in general, due to the poor distribution of health services and *lack of planning*. I believe that perhaps [mentions municipality] could be self-sufficient in providing healthcare to the entire population if there were *planning and organization.* If there were, in fact, equity in the distribution of resources. […] what we see is that policy is made like this: "*Which neighborhood, I don't know, has the most organized Councils*?" They get more. Those where the Councils are weaker don't get it as easily" (Interview-BHU16).

BHU: basic health unit; FG: focal group.

The discourses pointed to weaknesses in communication flows between the health secretariat, regional management, and BHU, as well as weaknesses in the criteria for defining testing in BHU, with repercussions on the differentiated functioning of the BHU and perceived by the interviewees in the need for the teams to construct such definitions (S14, S15, S16 — Chart 3) — thus operationalizing elements of micro-management^16^.

The municipality established regulations; however, the speed of dissemination and magnitude of the pandemic would lead to a possible mismatch in the time required to construct definitions and their dissemination. Added to this is the fragile national coordination in facing the pandemic^5,7^, which may have intensified the management challenges for the other spheres of SUS in the country.

It is interesting to note that in one of the statements (S17 — Chart 3), the perception of macro-management appears at the interface with social control in SUS.

The results therefore show that addressing the challenges reflected in the different components of the health system ultimately encounters the chronic underfunding of SUS, accentuated by fiscal austerity measures such as Constitutional Amendment No. 95/2016 — known as the "Spending Cap"^29^.

The Covid-19 pandemic confirmed the importance of the SUS as a public and universal system. The threats to the SUS and its constitutional principles must be confronted, and its defense must be woven into all areas of society. It is also necessary to strengthen PHC with a view to a more effective system and the development and implementation of a "democratic SUS"^29^.

Finally, it is worth noting that the period in which the data was collected (during the circulation of the Omicron variant) may have contributed to the fact that important events and other challenges from the beginning of the pandemic were not recalled. The number of interviewees is also a limitation of the study, so the narratives reflect the experiences of some professionals and may not express the perception of all professionals in the municipality.

## CONCLUSION

Reports on the implementation of testing in BHU revealed professionals who, amidst many challenges, managed to incorporate new practices. Limitations in human resources, supplies, and the physical structure of the BHU are some of the challenges identified for the widespread implementation of Covid-19 testing in these units, reinforcing the need to increase investments in infrastructure and advance actions to strengthen PHC and value its workers.

Despite the challenges and some deviations from best practices, perceptions regarding the need for widespread testing in BHU were prominent. The findings point to the importance of coordinating the pandemic response and effectively decentralizing the actions adopted, developing preparedness plans for health crises, professional training programs, and improving communication flows between management and teams. Furthermore, actions focused on worker health are necessary.

The scarcity of personal protective equipment and other supplies for confronting the pandemic, especially in the initial period, highlighted inequalities in the capacity for acquisition and production among countries — which, from a macro perspective, exposed the urgency of building strategies to strengthen the health economic-industrial complex in the country. The pandemic also revealed the importance of improving occupational health programs and, fundamentally, strengthening and defending SUS.

It is suggested that studies be developed to report the results achieved in confronting the pandemic in different PHC contexts and the respective strategies for implementing testing.

## Data Availability

The study data not included in the article are not available.
