# Supplementary material for: Covid-19 testing in Primary Health Care: professionals’ perceptions
Source: Rev Saude Publica. 2026 May 1;60(Suppl 1):e7s. doi: 10.11606/s1518-8787.2026060006430 (PMC13138123; doi:10.11606/s1518-8787.2026060006430)
Supplement: Translation

# Translation: Testagem para Covid-19 na Atenção Primária à Saúde: percepção dos profissionais

**Authors:** Denise Nogueira Cruz, Sandra Garrido de Barros, Ana Clara de Rebouças Carvalho, Thais Regis Aranha Rossi, Laio Magno, Inês Dourado

**Affiliations:** I Universidade Federal da Bahia. Faculdade de Odontologia. Departamento de Odontologia Social e Pediátrica. Salvador, BA, Brasil; II Universidade Federal da Bahia. Instituto de Saúde Coletiva. Salvador, BA, Brasil; III Universidade do Estado da Bahia. Departamento de Ciências da Vida. Salvador, BA, Brasil; IV Fundação Oswaldo Cruz. Instituto Gonçalo Moniz. Salvador, BA, Brasil

**Keywords:** COVID-19, Atenção Primária à Saúde, Serviços de Saúde, Testagem

## Abstract

**OBJETIVO:**

Analisar percepções de profissionais da Atenção Primária à Saúde (APS) sobre a testagem para Covid-19 em unidades básicas de saúde (UBS).

**MÉTODOS:**

Estudo qualitativo, integrante de pesquisa formativa, envolvendo 22 entrevistas semiestruturadas e 4 grupos focais com profissionais que atuavam na APS de uma capital brasileira. Selecionou-se, entre outros critérios, o profissional de cada UBS mais diretamente envolvido com ações de enfrentamento à pandemia. Foi realizada análise de conteúdo com abordagem analítica dedutiva em que os achados foram organizados em categorias definidas com base nos componentes do sistema de saúde (população; infraestrutura; organização dos serviços; prestação dos serviços ou modelo de atenção; gestão).

**RESULTADOS:**

Relatos comumente referiram, no componente população, os desafios procedentes dos limites de cobertura da APS; na infraestrutura, destacou-se a insuficiência de recursos humanos e infraestrutura física que, por sua vez, exigiram mudanças na organização e na prestação de serviços, com reflexos no modelo de atenção. As contribuições revelaram, ainda, que em alguns momentos da pandemia houve descaracterização do processo de trabalho, especialmente nas equipes de saúde da família. Pouco identificado, no componente gestão foram apontadas fragilidades nos fluxos de comunicação entre secretaria de saúde e UBS.

**CONCLUSÃO:**

Constatou-se a percepção de que a ampliação e capilarização dos testes nas UBS eram necessárias. Os achados apontam para a importância da coordenação da pandemia e descentralização efetiva das ações adotadas, de planos de preparo e fortalecimento da Atenção Primária à Saúde e dos seus profissionais, bem como investimentos em infraestrutura e capacitação das equipes e avanços nas ações voltadas à saúde do trabalhador. A pandemia desvelou a importância de avançar no complexo econômico-industrial da saúde e, fundamentalmente, fortalecer e defender o Sistema Único de Saúde brasileiro.

## INTRODUÇÃO

A pandemia de Covid-19 teve repercussões em diferentes áreas da vida humana e exigiu mudanças na organização e oferta dos serviços de saúde em escala global. A ausência inicial de vacinas e outras medidas farmacológicas implicou a adoção de ações de contenção e mitigação a serem complementadas por estratégias de testagem e rastreio^1^. Contudo, a testagem e o diagnóstico, nos primeiros estágios da pandemia, representaram um desafio em razão de dificuldades que incluíam aspectos relativos ao material biológico a ser utilizado, à metodologia a ser empregada, à acurácia dos testes e à disponibilidade de insumos e recursos dada a demanda mundial^2^. Assim, o desenvolvimento e a disponibilidade dos testes rápidos de antígeno conformavam uma estratégia de expansão do acesso à testagem e de enfrentamento à pandemia pela possibilidade de diagnosticar casos ativos e impedir a propagação do vírus^3^.

No contexto inicial da pandemia, o governo federal brasileiro priorizou a testagem em hospitais e estabelecimentos de saúde para indivíduos sintomáticos^4,5^, e a ausência de uma estratégia nacional coordenada e abrangente para testagem e rastreamento acentuou os desafios de abastecimento de insumos para diagnóstico e de contenção da disseminação do vírus^4,6^. Por outro lado, destaca-se a articulação de governos locais e outros atores sociais em torno de iniciativas para aumentar a capacidade de testagem e produção nacional de componentes, a exemplo do aprimoramento dos testes PCR; o desenvolvimento de tecnologias; e a implementação de medidas regulatórias para acelerar novos testes^4^.

No Brasil, a existência de um sistema público e universal de saúde, o Sistema Único de Saúde (SUS), foi fundamental no enfrentamento da pandemia^7^ — que exigia (re)estruturação da atenção especializada, a exemplo da ampliação de leitos hospitalares, mas também da Atenção Primária à Saúde (APS). Contudo, como em outros países, houve uma baixa priorização da APS^5^, o que, mesmo tendo desempenhado papel relevante^8^, pode ter limitado seu potencial tendo em vista sua capilaridade no território brasileiro. Provavelmente, o amplo percentual de cobertura da APS viabilizaria o fortalecimento das ações de vigilância na resposta à Covid-19, com base na abordagem comunitária e territorial, e permitiria potencializar a atuação dos agentes comunitários de saúde (ACS), especialmente nas situações de pouca capacidade diagnóstica e de testagem^5^.

A fragilidade na articulação entre vigilância em saúde e APS na resposta brasileira e os baixos níveis de testagem, em período inicial da pandemia, dificultaram as ações de monitoramento da situação epidemiológica^7^. No que diz respeito às ações de vigilância em saúde, uma investigação em municípios de grande porte do Nordeste brasileiro constatou incipiência nas ações de busca^9^; já no estudo com unidades básicas de saúde (UBS) do país, os piores resultados, nas cinco regiões brasileiras, foram relacionados à coleta de testes de diagnóstico^8^.

Por outro lado, há estratégias com desfechos positivos, como a iniciativa de gestores municipais e gerentes de APS para estabelecimento de articulação regional e parceria com universidades, que possibilitaram ampliação da testagem^10^.

Durante a pandemia, as práticas dos profissionais da APS foram modificadas, e a literatura tem registrado estudos de percepção destes profissionais sobre o que são entendidas como situações de crise e como gerenciá-las^11^, sobre o papel da APS no enfrentamento da pandemia^12^, e no tocante à campanha de vacinação contra a Covid-19^13^. Entretanto, pelo nosso conhecimento, a percepção dos profissionais quanto à testagem para Covid-19 ainda não foi explorada. O fim do estado de emergência da pandemia foi decretado em maio de 2023^14^, e as evidências acumuladas interpelam a produção de investigações que contribuam para informar respostas na gestão de futuras crises sanitárias.

Assim, o objetivo deste estudo foi analisar percepções de profissionais que atuavam na APS sobre a testagem para Covid-19 em UBS de uma capital brasileira.

## MÉTODOS

Trata-se de um estudo de abordagem qualitativa, com dados oriundos da Pesquisa Formativa (PF) do projeto "Expansão das estratégias de testagem, quarentena, *e-health* e telemonitoramento para contribuir no enfrentamento da pandemia de Covid-19 no Brasil — TQT Covid-19", desenvolvido em dois municípios brasileiros. A PF buscou compreender o processo de trabalho nas UBS — com e sem a Estratégia de Saúde da Família (ESF) —, bem como os desafios relativos à realização da testagem de Covid-19 nas unidades, de modo a apontar estratégias mais adequadas para sua implementação e expansão. Embora realizada nos dois municípios, este estudo trata dos dados procedentes da PF de um dos municípios.

Foram realizadas 22 entrevistas semiestruturadas e 4 grupos focais (GF) com 23 profissionais de diferentes categorias que atuavam em uma das 22 UBS, com e sem ESF, envolvidas no estudo e de uma região territorial populosa e com elevada vulnerabilidade social de uma capital brasileira. Considerando a participação de duas profissionais em ambas as estratégias (GF e entrevista), o estudo envolveu 43 profissionais.

Os dados foram produzidos entre dezembro de 2021 e março de 2022, ressaltando que nesse período a testagem não era realizada em todas as UBS. As entrevistas foram realizadas presencial e individualmente com um(a) profissional de cada UBS selecionado(a) com base na indicação da gerência das UBS (conveniência); disponibilidade; que estivesse mais diretamente envolvido(a) nas ações de enfrentamento da pandemia (intencionalidade); e com pelo menos um ano de experiência na função exercida (elegibilidade). Os GF foram constituídos por profissionais de algumas UBS que realizavam testes para Covid-19 e outras que não realizavam testagem, e pelos(as) respectivos(as) gerentes das UBS. Um GF foi formado apenas por ACS de diferentes UBS. As entrevistas e os GF foram conduzidos por pesquisadoras que compunham a pesquisa formativa — e nos GF, especificamente, houve presença de mais de uma pesquisadora para garantir a moderação e o apoio na condução.

O roteiro das entrevistas abordava questões relativas à pandemia de Covid-19; às medidas de enfrentamento e ao papel da APS; à organização das ações de testagem, à vacinação e vigilância dos casos nas UBS; ao conhecimento sobre testes para Covid-19; às medidas de proteção social e fonte de informações/notícias da pandemia. O roteiro dos GF tinha como temática central a testagem para Covid-19, incluindo questões sobre condutas, os critérios e as dificuldades para realização dos testes nas UBS; treinamento e conhecimento dos profissionais, buscando, pela interação grupal e pelos diferentes pontos de vista, apreender percepções e práticas em torno da testagem.

As entrevistas (variando de 29 a 104 minutos) e os GF conformaram, respectivamente, 23h19min e 5h47min de gravações que foram transcritas na íntegra, revisadas e codificadas. Após a transcrição, foi procedida a análise de conteúdo, incluindo as etapas de pré-análise, a sondagem do material e o tratamento, a inferência e interpretação dos resultados^15^. A análise visou compreender as percepções acerca da testagem nas UBS, articulando-as com os componentes de um sistema ou de uma rede de serviços de saúde^16^.

Buscou-se apreender a realização da testagem por meio dos relatos profissionais; de "histórias concretas"^17^, atento ao fato de que trazem a experiência vivida enquanto experiência subjetiva, dadas as suas diferentes trajetórias e relações com o evento^18^.

A análise foi realizada de acordo com categorias sobre os componentes de um sistema de saúde adotados por Souza e Bahia^16^, tendo sido aplicados cinco dos componentes (população; infraestrutura; organização dos serviços; prestação dos serviços ou modelo de atenção; e gestão), conforme Quadro 1. Com base nos elementos constituintes dos componentes do sistema de saúde, foram estabelecidas as seguintes categorias analíticas: população sob cuidado/adscrição; recursos humanos/trabalhadores; insumos; estrutura física/UBS; conhecimento/habilidades sobre testagem; ações de assistência, vigilância e continuidade de programas da APS; lógica/modelo de organização das ações nas UBS; normatização/regulação das práticas; elementos da macro e microgestão.

**Quadro 1:** Síntese da caracterização dos componentes de um sistema ou de uma rede de serviços de saúde adotados por Souza e Bahia^16^ e utilizados para a análise dos dados.

SISTEMA DE SAÚDE OU REDE DE SERVIÇOS DE SAÚDE	COMPONENTES
1) População	Populações que estão sob responsabilidade sanitária das redes de atenção; das equipes de saúde.
2) Infraestrutura	Inclui vários tipos de recursos que podem ser classificados em: Trabalhadores da saúde; Estabelecimentos/infraestrutura física da rede de saúde; Medicamentos, equipamentos e outros insumos; Conhecimento (do estado de saúde das pessoas, das tecnologias de intervenção sobre a saúde e a doença, e do funcionamento dos serviços).
3) Organização dos serviços de saúde	Como os recursos humanos e tecnológicos são organizados para realizar as ações de saúde. Inclui, no âmbito do SUS: Assistência à saúde; Vigilância em saúde; Políticas e programas especiais; Política de humanização da atenção.
4) Prestação de serviços ou modelo de atenção à saúde	Inclui ações de promoção da saúde; de prevenção, diagnóstico e tratamento de doenças e agravos; e de reabilitação da saúde. Podem desenvolver-se fundamentadas em diferentes lógicas ou racionalidades, configurando diferentes modelos de atenção: Modelo hegemônico (médico-assistencial privatista); Modelo sanitarista; Modelos alternativos (p. ex.: vigilância da saúde, promoção da saúde e as cidades saudáveis, modelo em defesa da vida e as ações programáticas de saúde, entre outros).
5) Gestão	A gestão articula-se com os conceitos de governança ("ato de bem governar") e de regulação (leis, regras, normas e instruções). A gestão pode ser categorizada de acordo com: Níveis de gestão: macrogestão, mesogestão e microgestão; Dimensões: política, técnica e administrativa (em cada nível).

Nota: os autores adotam, ainda, um sexto componente, o financiamento (não aplicado neste estudo).

Fonte: Souza e Bahia - síntese do texto apresentado entre as páginas 53–60^16^.

O projeto foi aprovado pelo Comitê de Ética em Pesquisa, sob o número 53844121.4.1001.5030, e pelo Comitê de Ética em Pesquisa da Organização Mundial da Saúde (CERC.0128A e CERC.0128B), e os profissionais concordaram em participar por meio da assinatura do Termo de Consentimento Livre e Esclarecido (TCLE).

## RESULTADOS E DISCUSSÃO

O estudo envolveu 43 profissionais que, em sua maioria, eram do sexo feminino, autodeclararam-se negros(as) ou pardos(as), enfermeiros(as) e com idade entre 30 e 45 anos.

### População

A população, segundo Souza e Bahia^16^, é o principal componente de um sistema de saúde, devendo ser territorialmente definida de modo que as redes de saúde e equipes (especialmente da ESF) respondam às necessidades de saúde e assumam a responsabilidade sanitária por tal.

Os resultados obtidos revelaram que havia uma percepção dos participantes do quão importante é a adscrição da população e definição de território na organização dos serviços na APS.

"[…] a procura é muito grande, nem todas as unidades fazem. […] *todas as unidades deveriam fazer o teste. Até pela questão da facilidade de chegar à unidade […] o paciente muitas vezes vem andando para a unidade*. Ele não vem de transporte, ele não tem dinheiro para pagar a passagem de ônibus. Então, *se tiver perto de casa fica mais fácil*. Muita gente não faz teste porque não tem como chegar. […] Então, muita gente passa com a doença sem nem ser atendido, sem nem ter assistência" (Entrevista-UBS19).

Para os profissionais de equipes com e sem ESF, a realização de testes em todas as UBS melhoraria a oferta e reduziria as barreiras enfrentadas pela população.

"[imagine] eu estou com suspeita de Covid, estou na minha casa isolada, *mas tem um posto aqui no meu bairro perto. Eu vou ali e vou fazer o teste e volto para minha casa.* Mas, não… Só tem naquele posto lá longe que eu tenho que pegar um transporte […] e isso já vai contaminando outras pessoas, né? […] Deveria ter mais unidades de testagens. Cada bairro deveria ter um local de testagem para facilitar a população e eles não terem que se deslocar do local" (Entrevista-UBS20).

A APS é, preferencialmente, o primeiro contato dos indivíduos com o sistema de saúde, configurando-se no centro de comunicação de uma rede de atenção e ordenadora do cuidado^19^. Sistemas integrados de saúde^16^ que fortalecem a APS com definição de uma população adscrita são mais efetivos^19^. A organização da APS com implantação de UBS adequadas e suficientes, com oferta de serviços que respondam às necessidades dessa população e que sejam territorialmente mais próximas, pode reduzir barreiras financeiras, geográficas e organizacionais^20^ como as percebidas pelos entrevistados.

### Infraestrutura

É o conjunto de recursos necessários para a prestação dos serviços, incluindo: trabalhadores da saúde (atentando para quantitativo, competências, distribuição, treinamento e condições de trabalho); estabelecimentos/infraestrutura física da rede de saúde; medicamentos/equipamentos/insumos; e conhecimento (do estado de saúde da população, das tecnologias, do funcionamento dos serviços)^16^.

No domínio do componente "infraestrutura", os relatos apontaram que garantir a testagem nas UBS esbarrava em desafios relacionados aos quatro tipos de recursos (trabalhadores, insumos, estrutura física e conhecimento). Os achados e a análise sobre infraestrutura (para cada tipo de recurso) são apresentados a seguir, e os excertos das falas estão consolidados no Quadro 2 ao final deste componente.

**Quadro 2:** Excertos de falas das entrevistas individuais e dos grupos focais relacionadas às categorias estabelecidas para o componente infraestrutura.

Recurso - Trabalhadores	Fala 1 (F1)	"[…] a questão de ter a testagem aqui é a questão *pessoal, a gente não tem recursos humanos para isso*. […] Se a gente colocar mais uma coisa, a gente talvez não tenha como dar conta. […] Se tivesse a testagem, não ia ter a vacina. […] tem que ver o profissional que faz, a capacitação, quem que pode fazer, os horários de atendimento, se teria que ser todo dia. Teria que montar toda uma estratégia" (Entrevista-UBS5).
	Fala 2 (F2)	"[…] foi uma das grandes dificuldades que nós tivemos quando começou a fazer a testagem aqui: justamente, era a *falta de um médico* para prescrever e encaminhar" (Entrevista-UBS18).
	Fala 3 (F3)	"[…] nessa última semana aí […] a unidade teve que fechar e foi suspenso os testes. Exatamente por *condições de funcionário* mesmo. A *equipe [*…*] positivada*. […] não tinha condições, não teve como repor outro enfermeiro, não teve como repor o funcionário da higienização. […] *está suspenso o teste* até a segunda ordem" (Participante5-GF02).
Recurso - Insumos	Fala 4 (F4)	"[…] eu acho que *se tiver mais testes disponíveis, a gente consegue atender uma demanda maior* […] porque se a gente não recebe, aí a gente acaba limitando realmente […]. *Muitas pessoas até têm os sintomas, mas não procuram as unidades para fazer pela dificuldade de conseguir*, porque às vezes a unidade só faz uma quantidade, e tal, e aí acaba que a pessoa não procura o serviço, e fica lá: ‘Eu vou me tratar em casa sozinho’" (Participante4-GF04).
	Fala 5 (F5)	"*É angustiante!* Você vê uma fila de 30, 40 pessoas e só fazer 12 testes é *angustiante*!" (Participante1-GF01).
	Fala 6 (F6)	"Para as pessoas terem acesso, o que eles [referindo-se ao município] implementaram […]? Tirou o teste […] só de dentro das unidades de saúde, né? Para botar nesses locais móveis, né? […] *Não é numa unidade de saúde, é na rua, né? Vai uma unidade móvel*… Vai chegar lá, para e faz a testagem" (Entrevista-UBS20).
	Fala 7 (F7)	"Ampliar ou fazer quase todos os postos, como tem o teste rápido de doenças transmissíveis sexualmente. Então, *poderia ter em todos os postos, né? A testagem de Covid.* E, na época em que estiver no pico da pandemia, *disseminar para supermercado, para shoppings*" (Entrevista-UBS12).
Recurso - Infraestrutura das UBS	Fala 8 (F8)	"Aqui a gente não queria atender sintomático por conta disso. Porque *você está misturando dois públicos*. Você está misturando o público saudável com o público doente. […] a pessoa que vem aqui saudável para tomar vacina acaba cruzando com essa pessoa que já está com sintoma e corre o risco de sair daqui doente. *A gente não tem estrutura física para isso.* Se fosse *uma unidade maior, com duas portas, uma porta de entrada e uma porta de saída*…" (Entrevista-UBS05).
	Fala 9 (F9)	"[…] *o certo não era ser dentro de uma unidade básica*. […] como é que você vai botar um paciente que está sintomático para fazer dentro de uma unidade onde tem farmácia, onde tem vacinação, onde tem […] todos os tipos de serviços […]. *Como é que você vai chegar na fila da farmácia e vai explicar* às pessoas […] *que aquele corredor de risco*, que é de Covid, entendeu?" (Entrevista-UBS06).
Recurso - Conhecimento	Fala 10 (F10)	"[…] que eu saiba, *nenhum de nós foi capacitado* para nenhuma das duas testagens [PCR e teste rápido] […]. Quando iniciou, […] não era nenhum de nós que fazia" (Entrevista-UBS01).
	Fala 11 (F11)	"[…] aprendemos no tombo […]. A gente não recebeu capacitação, nenhuma […]. Na minha unidade, a gente sentava e conversava, né? […] mas, assim, *eu mesmo não passei por capacitação*" (Participante3-GF03).
	Fala 12 (F12)	Participante 2: "Eu fui [capacitado] por ela […]. *Ela foi minha professora*". Participante 5: "Eu não, *o colega que aprendeu que me ensinou*". Participante 4: "*Só alguns técnicos*, e a gente tem uma moça que trabalha no laboratório. Só eles que tiveram" (Participantes 2, 4, 5-GF01).

UBS: unidade básica de saúde; GF: grupo focal.

A insuficiência de trabalhadores, percebida tanto nas UBS que realizavam testagem para Covid-19 quanto naquelas que não testavam, associava-se aos desafios de incorporar a testagem e garantir demais ações inerentes à APS, conforme revela a Fala 1 (F1) no Quadro 2. Os desafios, para além do quantitativo de recursos humanos, incluíam, ainda e por razões variadas, a ausência de algum profissional (F2, F3 — Quadro 2).

A limitação de insumos restringia a realização dos testes e ainda era percebida como fator resultante da não procura do serviço pela população e gerador de angústia nos entrevistados (F4, F5 — Quadro 2).

Importante também observar na F4 o contraste com a insuficiência de trabalhadores. Descortina-se uma outra percepção da experiência de testagem nas UBS, prevalecendo a disponibilidade de testes enquanto desafio.

A angústia proferida na F5 traduz o sofrimento do profissional resultante de uma demanda por testes maior do que a disponibilidade na UBS. A pandemia, por várias razões, desencadeou adoecimento psíquico nos profissionais de saúde da APS^21^. Tais achados devem provocar, e não apenas no município, o desenvolvimento de programas robustos voltados à saúde do trabalhador.

Uma percepção recorrente foi a necessidade de descentralização da testagem para todas as UBS e outros espaços sociais (F6, F7 — Quadro 2); medida que ampliaria o acesso diagnóstico da população, mas que tinha na insuficiência de insumos uma considerável limitação.

A escassez de testes para Covid-19 no mercado internacional nos primeiros estágios da pandemia resultou na definição de prioridades para sua realização e implementação de estratégias como "centros de testagem" e o sistema de *drive-through* — estas também incorporadas na Coreia do Sul^22^. Este país, assim como a China, foi exitoso na resposta à pandemia; ambos apostaram em medidas não farmacêuticas e investiram na expansão da testagem^22,23^.

As limitações de infraestrutura física das UBS foram descritas por muitos entrevistados como obstáculos na garantia da oferta simultânea de testes e vacina para Covid-19 (F8, F9 — Quadro 2). O tamanho das UBS; espaço de circulação interna; dimensionamento dos fluxos de entrada e saída de usuários; e o posicionamento de consultórios, setores e farmácia são algumas das dificuldades apontadas e que requerem atenção dos gestores no planejamento e na construção de estabelecimentos, supondo futuras crises sanitárias.

As considerações sobre a infraestrutura das UBS e a importância de sua implantação ser próxima às respectivas populações adscritas suscitam reflexões sobre os desafios relativos ao território geográfico, especialmente em áreas densamente populosas de grandes centros urbanos. Garantir área para a instalação de UBS territorial e estruturalmente adequadas torna-se um desafio, para além dos recursos humanos e financeiros, que deve ser problematizado em diferentes espaços — como aqueles de discussão dos planos de desenvolvimento municipais.

Acerca do conhecimento, outro tipo de recurso, foi recorrente, nos relatos sobre capacitação profissional para realização dos testes nas UBS, menção aos processos de capacitação pontual ou mesmo sua inexistência (F10, F11 — Quadro 2), bem como à estratégia de replicação das orientações por algum colega capacitado (F12 — Quadro 2). Contudo, a maioria percebia-se preparada para realizar a testagem.

Cabe registrar que o município oportunizou treinamento em serviço para teste rápido de antígeno por meio dos profissionais do laboratório central municipal, aspecto sinalizado na F12. Os discursos, contudo, não indicaram o emprego de tecnologias digitais ou estratégias para formação, especificamente, em torno da testagem — fato que chama a atenção, dada a expansão, a partir da pandemia, do uso de novas tecnologias digitais com aplicação, por exemplo, na vigilância da saúde^24^.

### Organização dos Serviços de Saúde

Este componente define como se organiza a assistência, a vigilância em saúde, as políticas e os programas especiais, além da política de humanização com base nos recursos humanos e tecnológicos disponíveis^16^.

Importante reparar que os limites envolvendo os componentes do sistema de saúde até aqui apresentados, especialmente infraestrutura das UBS, engendraram percepções relativas às dimensões da organização dos serviços e, também, da/do prestação/modelo de atenção (constatadas nos relatos adiante).

Os desafios da cobertura da APS, da infraestrutura das UBS e a insuficiência de testes/insumos implicaram negativamente nas ações de assistência à saúde da população e na continuidade dos programas realizados anteriormente, e ainda foram percebidos como entraves para articulação com as ações de vigilância em saúde.

"[…] a gente *não tem uma boa cobertura de atenção primária*, logicamente a *gente não vai ter uma boa cobertura de testagem* […] na minha opinião, *testagem não é só para paciente que está com sintomas*. A gente faz testagem em um paciente sintomático, mas aquele paciente que está assintomático está ali passando para todo mundo" (Participante1-GF4).

"[…] a gente concentra muito […] e a gente não capilariza. Então *isso gera confusão para quem tem um processo de trabalho* […] a ideia é que as ações da Covid, ao longo do tempo, ela faça parte do rol do processo de trabalho das equipes. Então, *quando você concentra, a equipe de certa forma para um pouco o seu processo de trabalho*, e isso *penaliza as outras condições que a equipe já trabalhou.* Então, se você pulveriza, você tem condição de diminuir essa sobrecarga, e as equipes tocarem mais seu trabalho. Porque *na unidade que faz teste, então para isso, para aquilo* […] você penaliza mais aquela unidade que está ofertando o serviço" (Participante2-GF4).

"*Eu tenho que ter o teste para fazer* […]. *Sem o teste, eu não tenho uma visão* […] *e o que me dá respaldo para fazer intervenção é investigação epidemiológica*. Se eu não faço investigação epidemiológica, eu fico no meu achismo. E, no meu achismo, eu vou pegar pontos de incêndios e não vou apagar incêndio todo" (Entrevista-UBS21).

### Modelo de Atenção

Refere-se às "combinações de tecnologias materiais e imateriais utilizadas nas intervenções sobre problemas e necessidades de saúde"^16^. Compreende modelos hegemônicos e modelos alternativos^25^.

Cabe anunciar que o entendimento aqui é de que a lógica norteadora das práticas das equipes de APS, especialmente da ESF, deve ser na direção dos "modelos de atenção alternativos"^25^. Enquanto proposta de redefinição de práticas e reorganização dos serviços, a vigilância da saúde preconiza a articulação das ações de controle de determinantes, riscos e danos à saúde, com abordagem individual e coletiva, e atuação em territórios, para enfrentamento de problemas e melhoria das condições de vida da população^25,26^. Esse modelo supera a dicotomia entre vigilância epidemiológica e sanitária ("práticas coletivas") e assistência ambulatorial e hospitalar (práticas individuais), integrando-as e possibilitando, ainda, articulação da demanda espontânea à oferta organizada por grupos populacionais na concepção das ações programáticas de saúde^26,27^.

No curso dessa discussão, destaca-se que os relatos dos profissionais revelaram dilemas e tensões diante da percepção: da descaracterização das práticas durante a pandemia, principalmente na fase inicial e nos momentos de recrudescimento dos casos; da descontinuidade no acompanhamento de determinados programas; e da ausência de uma agenda "programática".

"Nesse período mais intenso de acolhimento aos sintomáticos respiratórios, nós estávamos, praticamente, *fechados às demandas internas de programas* […]. Então o espaço da unidade ficou voltado a essa demanda […] que era o acolhimento ao sintomático respiratório" (Entrevista-UBS14).

"[…] na verdade, eu só via Covid! Depois de um tempo, eu comecei a ver doenças crônicas […] *doenças crônicas que perderam acompanhamento* porque suspenderam o ambulatório […]. Então, doenças crônicas que tinham controle, que estavam compensadas, descompensando num sistema superlotado" (Entrevista-UBS17).

"*Eletivo foi praticamente suspenso*… Tuberculose a gente continuava atendendo […]. Se fosse hipertenso, diabético descompensado, todo mundo conseguia ser atendido […]. Nas ondas, em todas as subidas e descidas de onda, a gente viveu assim. Sempre que *a gente via que reduzia a quantidade de sintomático, aí a gente ia reduzindo os turnos de equipe exclusiva para sintomático e abrindo a agenda*. Então a gente viveu assim, vive assim ainda, né? […] a gente vive observando a onda […]. *Então, sempre que aumenta, a gente já fica tenso*: ‘Vai ter que mudar […]’. A gente vive numa *tensão o tempo inteiro*" (Entrevista-UBS03).

A pandemia afetou rapidamente os sistemas de saúde, com impactos no processo de trabalho das equipes da APS, como apontam os achados acima. Os impactos na APS foram verificados nas diferentes fases da pandemia^28^, como suspensão inicial de atividades essenciais e de rotina e, em seguida, a retomada de algumas práticas conjugadas com a incorporação de ações — por exemplo, a testagem —, confluindo para descaracterização do processo de trabalho da APS^28^.

### Gestão

Este componente refere-se a como, nos diferentes níveis (macro, meso e microgestão), são desenvolvidas ações e formulações nas dimensões política, técnica e administrativa nos sistemas de saúde^16^. A gestão articula-se com conceitos de governança e de regulação^16^.

O componente da gestão foi eventualmente mencionado, mas aspectos relativos à regulação, especificamente definição de normas e instruções ou mesmo a fragilidade desses mecanismos, foram notados (F13 — Quadro 3).

**Quadro 3:** Excertos de falas das entrevistas individuais e grupos focais relacionadas às categorias estabelecidas para o componente gestão.

Gestão – normas/regulação	Fala 13 (F13)	"[…] se existe uma pandemia, a gente sabe que a gente vai ter que atender, *que se providencie esse protocolo* desde a porta de entrada. O fluxo a gente veio receber três meses depois. ‘Ah, pegou todo mundo de surpresa’. Eu acho que, na saúde, certas coisas não pode ser surpresa. […] o que eu queria, na porta de entrada até aqui: as atribuições de cada um. […] a gente quem bolou tudo! *Três, quatro meses depois que chegou para gente*" (Participante1-GF01).
Microgestão	Fala 14 (F14)	"[…] um caos total […]. É aquilo que eu estava falando: *atravessou a rua, a unidade é de outro jeito*" (Entrevista-UBS03).
	Fala 15 (F15)	"São atendimentos diferentes […]. Ficou parecendo o seguinte: que *a gente fez estratégia de guerra* […] e nós vivemos uma coisa que a gente nunca viveu na vida; ninguém nunca viveu pandemia. Então, assim, *seria muito estranho se os colegas de um posto agissem igual a de outro posto*" (Participante 6-GF04).
	Fala 16 (F16)	R: […] cada unidade tem uma dinâmica. Mas, *minimamente, isso precisava ter um padrão*, né? Não tem. P: […] me lembrando da reunião […] falaram que uma unidade fazia 40 testes, a outra fazia 50, a outra fazia 10. Quem determina isso? R: Ninguém. É a unidade. A unidade em diálogo com o [refere gestão regional] que determina […]. Não tem *relação nenhuma com demanda*. O município deixa a cargo das equipes. […] aqui a gente teve vários tensionamentos" (Entrevista-UBS03).
Macrogestão	Fala 17 (F17)	"[…] o que dificulta no município […] não só o acesso ao teste, como o acesso à atenção primária e à atenção em saúde de uma forma geral, é a má distribuição dos serviços de saúde, *falta de planejamento*. Eu creio que talvez [cita município] fosse autossuficiente, realmente, na condição de fornecer assistência de saúde a toda população *se tivesse planejamento e organização*. Se tivesse, de fato, equidade na distribuição dos recursos. […] o que a gente vê é que *a política* é feita assim: "Quem é o bairro que, sei lá, *os Conselhos estão mais organizados?". Conseguem mais.* Aqueles onde os Conselhos são mais frágeis, não conseguem com tanta facilidade" (Entrevista-UBS16).

UBS: unidade básica de saúde; GF: grupo focal.

Os discursos apontaram fragilidades nos fluxos de comunicação entre secretaria de saúde, gestão regional e UBS, bem como fragilidades nos critérios para definição da testagem nas UBS, com reflexos no funcionamento diferenciado das UBS e percebidas pelos entrevistados na necessidade de as equipes construírem tais definições (F14, F15, F16 — Quadro 3) — operacionalizando, assim, elementos da microgestão^16^.

O município estabeleceu normativas, contudo, a velocidade de disseminação e magnitude da pandemia acarretaria um possível descompasso no tempo exigido para construção de definições e sua publicização. Acrescenta-se a frágil coordenação nacional no enfrentamento da pandemia^5,7^, que pode ter intensificado os desafios de gestão para as demais esferas do SUS no país.

Interessante notar que em uma das falas (F17 — Quadro 3) a percepção sobre a macrogestão aparece na interface com o controle social no SUS.

Os resultados evidenciam, portanto, que o enfrentamento dos desafios refletidos nos diferentes componentes do sistema de saúde depara, enfim, com o subfinanciamento crônico do SUS acentuado por medidas de austeridade fiscal a exemplo da Emenda Constitucional n.° 95/2016 — conhecida como "Teto de Gastos"^29^.

A pandemia de Covid-19 confirmou a importância do SUS enquanto sistema público e universal. As ameaças ao SUS e aos seus princípios constitucionais devem ser enfrentadas, e a sua defesa deve ser tecida em todos os espaços da sociedade. É necessário, ainda, fortalecer a APS com vistas a um sistema mais efetivo e ao desenvolvimento e à implementação do "SUS democrático"^29^.

Por fim, cabe sinalizar que o período da produção dos dados (já na circulação da variante Ômicron) pode ter contribuído para que fatos importantes e outros desafios do início da pandemia não fossem rememorados. O quantitativo de entrevistados também é um limite do estudo, de modo que as narrativas retratam vivências de alguns profissionais, podendo não expressar a percepção do conjunto de profissionais do município.

## CONCLUSÃO

Os relatos sobre a realização da testagem nas UBS revelaram profissionais que, em meio a muitos desafios, conseguiram incorporar novas práticas. Os limites de recursos humanos, insumos e na estrutura física das UBS são alguns dos desafios apontados para a capilarização da testagem para Covid-19 nas UBS, o que reforça a necessidade de ampliar investimentos na infraestrutura e avançar nas ações de fortalecimento da APS e valorização de seus trabalhadores.

A despeito dos desafios e de alguma descaracterização das práticas, sobressaíram percepções sobre a necessidade de capilarização dos testes nas UBS. Os achados apontam para a importância da coordenação da pandemia e descentralização efetiva das ações adotadas, de planos de preparo para crises sanitárias, programas de formação profissional, e avanços nos fluxos de comunicação entre gestão e equipes. Ainda, ações voltadas à saúde do trabalhador são necessárias.

A escassez de equipamentos de proteção individual e demais insumos para enfrentamento da pandemia, sobretudo em período inicial, estampou desigualdades na capacidade de aquisição e produção entre os países — o que, em uma perspectiva macro, expôs a urgência em construir direções para o fortalecimento do complexo econômico-industrial da saúde no país. A pandemia desvelou, ainda, a importância de aprimorar programas de saúde do trabalhador e, fundamentalmente, fortalecer e defender o SUS brasileiro.

Sugere-se o desenvolvimento de estudos que venham relacionar os resultados alcançados no enfrentamento da pandemia em diferentes contextos de APS e as respectivas estratégias de implantação da testagem.

## Data Availability

Os dados da pesquisa não inclusos no artigo não estão disponíveis.
